# Is Early Spatial Skills Training Effective? A Meta-Analysis

**DOI:** 10.3389/fpsyg.2020.01938

**Published:** 2020-08-27

**Authors:** Weipeng Yang, Haidan Liu, Nanxi Chen, Peng Xu, Xunyi Lin

**Affiliations:** ^1^S R Nathan School of Human Development, Singapore University of Social Sciences, Singapore, Singapore; ^2^Faculty of Education, East China Normal University, Shanghai, China; ^3^School of Education, University of Michigan, Ann Arbor, MI, United States; ^4^Faculty of Education, Beijing Normal University, Beijing, China; ^5^Faculty of Education, Victoria University of Wellington, Wellington, New Zealand; ^6^College of Education, Fujian Normal University, Fuzhou, China

**Keywords:** spatial skills, infancy and early childhood, training, meta-analysis, spatially enriched curriculum, STEM

## Abstract

Spatial skills significantly predict educational and occupational achievements in science, technology, engineering, and mathematics (STEM). As early interventions for young children are usually more effective than interventions that come later in life, the present meta-analysis systematically included 20 spatial intervention studies (2009–2020) with children aged 0–8 years to provide an up-to-date account of the malleability of spatial skills in infancy and early childhood. Our results revealed that the average effect size (Hedges's *g*) for training relative to control was 0.96 (*SE* = 0.10) using random effects analysis. We analyzed the effects of several moderators, including the type of study design, sex, age, outcome category (i.e., type of spatial skills), research setting (e.g., lab vs. classroom), and type of training. Study design, sex, and outcome category were found to moderate the training effects. The results suggest that diverse training strategies or programs including hands-on exploration, visual prompts, and gestural spatial training significantly foster young children's spatial skills. Implications for research, policy, and practice are also discussed.

## Introduction

Spatial skills are often applied in problem-solving situations, especially when processing and manipulating visuospatial information (Rafi et al., 2005). Studies have revealed that these skills strongly predict educational and occupational achievements in STEM (science, technology, engineering, and mathematics) domains (Wai et al., 2009; Lubinski, 2010; Uttal and Cohen, 2012; Stieff and Uttal, 2015). Improving spatial skills is therefore an important agenda for both research and educational practice (Hawes et al., 2017). Although previous studies have showed that early interventions for young children are more effective than interventions that come later in life (Heckman and Masterov, 2007), to what extent spatial skills training programs can effectively improve young children's spatial development remains understudied. It is worth noting that Uttal et al. (2013) have conducted a meta-analysis of training studies on spatial skills in general populations. However, this seminal work only included research evidence produced in 1984–2009 and did not focus on the training of early spatial skills.

In the past decade, we have observed an increase in research work on early spatial training and its effects, with more types of training approaches being used. In order to achieve an up-to-date understanding of the malleability of spatial skills in infancy and early childhood, the present meta-analytic study aims to synthesize spatial intervention studies that target young children aged 0–8 years from 2009 to 2020. Using a 2 × 2 typology of spatial skills (intrinsic vs. extrinsic and static vs. dynamic; Newcombe and Shipley, 2015), we meta-analyzed the eligible (quasi-)experimental studies for examining the effect of training on early spatial skills and the potential moderating effects on the relationship between the training and early spatial development.

### Spatial Skills in the Early Years

Spatial skills refer to the cognitive processing of spatial information, which “concerns shapes, locations, paths, relations among entities and relations between entities and frames of reference” (Newcombe and Shipley, 2015, p. 180). There are two traditions of conceptualizing spatial skills, including the psychometric approach and the classification system approach (Uttal et al., 2013). The former relies on exploratory factor analysis for identifying the key components of spatial skills, while the latter is rooted in a system comprised of two fundamental distinctions, i.e., between intrinsic and extrinsic information and between static and dynamic tasks (Uttal et al., 2013; Newcombe and Shipley, 2015). In this study, we extended the line of research on spatial skills training by following the 2 × 2 framework of spatial skills used in Uttal et al.'s (2013) seminal meta-analysis. According to Newcombe and Shipley (2015), the 2 × 2 typology of spatial skills leads to four categories of spatial skills and various assessments, as shown in Table 1. Based on the 2 × 2 framework of spatial skills (Newcombe and Shipley, 2015), the measurements of spatial skills can be put into categories as aligned with the four categories of spatial skills.

**Table 1:** The 2 × 2 typology of spatial skills and examples of each category.

**Category**	**Description**	**Example**	**Measurement**
Intrinsic–static	Configuration of objects	To categorize objects based on their spatial features	Mazes, Odd One Out Span, etc.
Intrinsic–dynamic	Transformation of the spatial codings of objects	To imagine the future state of rotating an object	Mental rotation test, Visual–Spatial Puzzle Task, etc.
Extrinsic–static	Identifying the spatial location of objects relative to others	To represent the location of objects in a map	Rod and Frame Test, performance of spatial relations, etc.
Extrinsic–dynamic	Transformation of the inter-relations of objects in movement	To enable perspective taking in understanding astronomy	Piaget's Three Mountains Task, water tilting task, etc.

*Newcombe and Shipley (2015); Uttal et al. (2013)*.

Spatial skills or spatial thinking skills are found to undergo considerable development during infancy and early childhood (0–8 years of age) (Newcombe and Frick, 2010). Prior research evidence indicated that infants as young as 4 months could show precursors of mental transformation (Rochat and Hespos, 1996; Hespos and Rochat, 1997). Frick and Wang (2010) also found that 13- to 16-month-old infants could perform mental rotation tasks after practice. Besides mental rotation, Bai and Bertenthal (1992) showed that 8-month-old infants had the ability of perspective taking when they moved to keep track of the location of an object. Preschoolers aged 3–5 years were also shown to be able to locate an object relative to a different viewpoint (Newcombe and Huttenlocher, 1992). However, individual differences exist in the early development of spatial skills (Hazen, 1982; Harris et al., 2013).

The significance of early spatial skills has been demonstrated by an extensive body of research, which links the early development of spatial thinking to map use (Liben et al., 2013), numerical skills (Zhang, 2016; Cornu et al., 2018; Fanari et al., 2019), arithmetic development (Zhang et al., 2014), math reasoning (Casey et al., 2015), math knowledge (Rittle-Johnson et al., 2019), early writing skills (Bourke et al., 2014), motor skills (Jansen and Heil, 2010), and executive functions (Lehmann et al., 2014; Frick and Baumeler, 2017). However, several lines of evidence suggest that there are early sex and socioeconomic status (SES) differences in spatial skills, with advantages for males and those with higher SES on spatial tests (Levine et al., 1999, 2005; Quinn and Liben, 2008). Therefore, it is of importance to know whether early spatial skills can be improved, especially in girls and socially disadvantaged children.

Neurological evidence supports that early intervention can enhance the neural functioning for spatial thinking (Gersmehl and Gersmehl, 2007). Prior studies also showed that the effects of early spatial training could be transferred to children's math skills (Cheng and Mix, 2014; Bower et al., 2020; Ribeiro et al., 2020; Thomson et al., 2020) and science understanding (Bower, 2017). For instance, Ribeiro et al. (2020) and Thomson et al. (2020) revealed that parental support such as spatial concept support and spatial language use in block building tasks or toy play situations tended to enhance young children's math performance. However, whether spatial skills training and support could lead to a substantial magnitude of improvement in early spatial development, as well as how it can be brought in an early childhood setting and incorporated into an early childhood curriculum, deserves more research.

### Malleability of Spatial Skills and Early Interventions

Previous research supports that spatial skills are malleable and can be improved through spatial training or instruction. However, most of the solid evidence for supporting the malleability of spatial skills is revealed by studies in the population of adolescents and adults (Uttal et al., 2013). In the most recent meta-analysis of spatial skills training studies conducted by Uttal et al. (2013), 217 intervention studies were included for analysis, revealing that the average effect size for spatial skills training relative to control was Hedges's *g* = 0.47 (SE = 0.04). However, of the 217 studies, only 53 studies focus on children younger than 13 years, with very few focusing on infants, toddlers, and preschoolers. Therefore, it remains to be further explored how to promote spatial skills in the early years.

It is worth noting that most of the training interventions were conducted in a much more controlled setting rather than the naturalist educational setting (Uttal et al., 2013; Hawes et al., 2017). Recent studies (e.g., Newcombe and Frick, 2010) have suggested that integrating spatial content into formal and informal instruction is meaningful for improving spatial functioning and reducing digital divides as related to sex and SES. As a result, more research is needed to test whether there is a difference in training effects across diverse settings, as well as demographic factors such as sex and SES. This will be a significant step forward in searching for an early spatially enriched curriculum (or “spatial curriculum” as promoted by Uttal, 2012) demonstrating the educational relevance of spatial training in the early years.

In terms of classroom-based spatial training, some have been conducted in early childhood settings. For instance, Ehrlich et al. (2006) found that gesturing provided meaningful cues about 5-year-old children's spatial strategies, which implied that gesture-based spatial training in the early childhood setting could be effective in improving mental rotation skills. In an experimental study, Casey et al. (2008) used block building activities to promote 6-year-old kindergarteners' spatial skills. They found that storytelling would provide a practical and useful context for teaching spatial content, while block building could develop children's various spatial skills (Casey et al., 2008). Petty and Rule (2008) also demonstrated the impact of mapping activities as supported by the use of materials such as toy figures, toy buildings, and photograph maps on the spatial skills of children aged 2.5–9, through a pretest–posttest quasi-experimental study. Furthermore, Hawes et al. (2015) conducted a randomized controlled trial among 6- to 8-year-olds to test the impacts of spatial skills training in regular classroom settings. Their research used iPad devices as the platform of early spatial skills training, and the intervention lasted 6 weeks. Evidence indicated that as compared to children in the control group, children who received the computerized spatial training demonstrated enhanced spatial skills (i.e., mental rotation) (Hawes et al., 2015). To make the spatial training more situated in the classroom, Hawes et al. (2017) further designed a 32-week geometry curriculum and conducted another experimental research study with 6-year-olds in their school. Results revealed that those young children's spatial and numerical skills (i.e., spatial language, visual–spatial reasoning, mental rotation, and symbolic number comparison) had been effectively improved using the spatially enriched approach to early geometry instruction (Hawes et al., 2017).

In the past decade, there have been an increasing number of studies on the effects of early spatial skills training. In general, these studies seem to support that young children would significantly benefit from participating in intentional spatial tasks or activities. However, the effects of early spatial skills training have not been systematically investigated. To address this knowledge gap, we conducted this meta-analytic study to examine the effects of interventions on spatial skills among children aged 0–8 years. This study intended to determine to what extent early spatial skills training would work and what the potential moderating factors are (e.g., study design, sex, age, category of spatial skills assessment, research setting, and type of training).

### The Present Meta-Analytic Review

As mentioned above, spatial skills are shown to be malleable; therefore, early spatial skills training activities comprised of interactive components such as hands-on exploration and environmental feedback (e.g., visual cues) are expected to show positive effects. This theoretical assumption can be further supported by understanding the early development of spatial skills (i.e., early spatial development).

The underlying mechanism of early spatial development is complex and dynamic, as comprised of multiple elements, including natural maturation, cultural scaffolding, environmental feedback, and active exploration (Newcombe and Learmonth, 1999). It involves both quantitative and qualitative aspects of cognitive change and continuity (Newcombe and Learmonth, 1999), which could be explained by Piaget's theory of cognitive development and Vygotsky's social development theory. The spatial development framework (Piaget, 1953; Piaget and Inhelder, 1956) describes children's progressive understanding of spatial relationships, from appreciating limited objects in the topological stage to considering distances and angles in the Euclidean stage. Although Piaget's cognitive constructivist approach has minimal emphasis on the role of cultural scaffolding, the functioning of schema through assimilation and accommodation provides implications that children's cognitive development can benefit from their interaction with the (physical) world in which they are living. Apart from Piaget, Vygotsky's (1978) sociocultural approach suggests that social interaction plays a fundamental role in cognitive development, which also applies to the specific development of spatial cognition.

Accordingly, the theoretical mechanism of early spatial development has assumed that environmental feedback and guidance in spatial training will improve an individual's ability to handle and manipulate specific spatial tasks. This meta-analysis assessed the extent to which spatial skills training programs could effectively improve young children's spatial development. Some meta-analytic or systematic reviews have examined the effectiveness of spatial skills training or related experiences (e.g., Baenninger and Newcombe, 1989; Spence and Feng, 2010; Uttal and Cohen, 2012; Uttal et al., 2013). However, to our knowledge, to date, there has been no systematic and dedicated research to examine the effect of spatial training on improving the spatial skills of children aged 0–8 years. To address this knowledge gap, we explored the effects of spatial skills training in the crucial life periods of infancy and early childhood, lasting from birth to 8 years. The following research questions thus guided this meta-analytic study:

1. What is the effect of early training on the spatial skills of children aged 0–8?
2. What variables moderate the effect of early spatial skills training?

## Methods

### Literature Search

The first author and the third author conducted an extensive automated search of electronic articles through the databases of PsycINFO, ERIC, EBSCO, ProQuest, and Scopus from February 1, 2009, through February 1, 2020. The literature search aimed to thoroughly identify randomized controlled trials or (quasi-)experiments studying the effects of early childhood interventions on the spatial skills development of children aged 0–8 years. Three different sets of terms with two Boolean operators (AND and OR) and the truncation character (^*^) were utilized to search for and download relevant literature from the databases: predictors (specific terms included “curriculum,” “intervention,” “approach,” “training,” and “program”), outcomes (specific terms included “spatial^*^,” “space,” “map,” “form perception,” “visual^*^,” and “visuospatial”), and sample (specific terms included “preschool,” “pre-K,” “prekindergarten,” “pre-kindergarten,” “kindergarten,” “primary school,” “elementary school,” “younger children,” “infant,” “toddler,” and “young children”). We created the search terms through extensive piloting. We used the operators “AND,” to connect search terms between the categories, and “OR,” to connect search terms within each category.

### Inclusion and Exclusion Criteria

Two researchers (the first two authors) independently selected and reviewed a subset (25%) of the articles following the inclusion criteria:

1. Included studies were (quasi-)randomized controlled trials or (quasi-)experimental designs.
2. Participants were 0–8 years of age (i.e., mean age of the participants).
3. Spatial skills were measured as outcomes of the intervention.
4. The reported information was sufficient enough for effect sizes to be calculated.
5. English was the written language used.

We excluded correlational studies (e.g., Levine et al., 2012) and reviews (e.g., Zimmermann et al., 2019). Non-full-text documents were also excluded because they may lack sufficient and credible information for meta-analysis.

### Study Selection

Based on the above inclusion and exclusion criteria, the two researchers divided 25% of the selected articles into three categories: eligible, possibly eligible, and ineligible. The inter-rater reliability was good (Cohen's kappa coefficient κ = 0.70) (Cohen, 1960). In view of the differences, the two researchers discussed the adequacy of the articles marked as “possibly eligible” and made the final inclusion decision based on full common consensus. The first author finished the selection of the remaining articles (75%).

**Figure 1:**
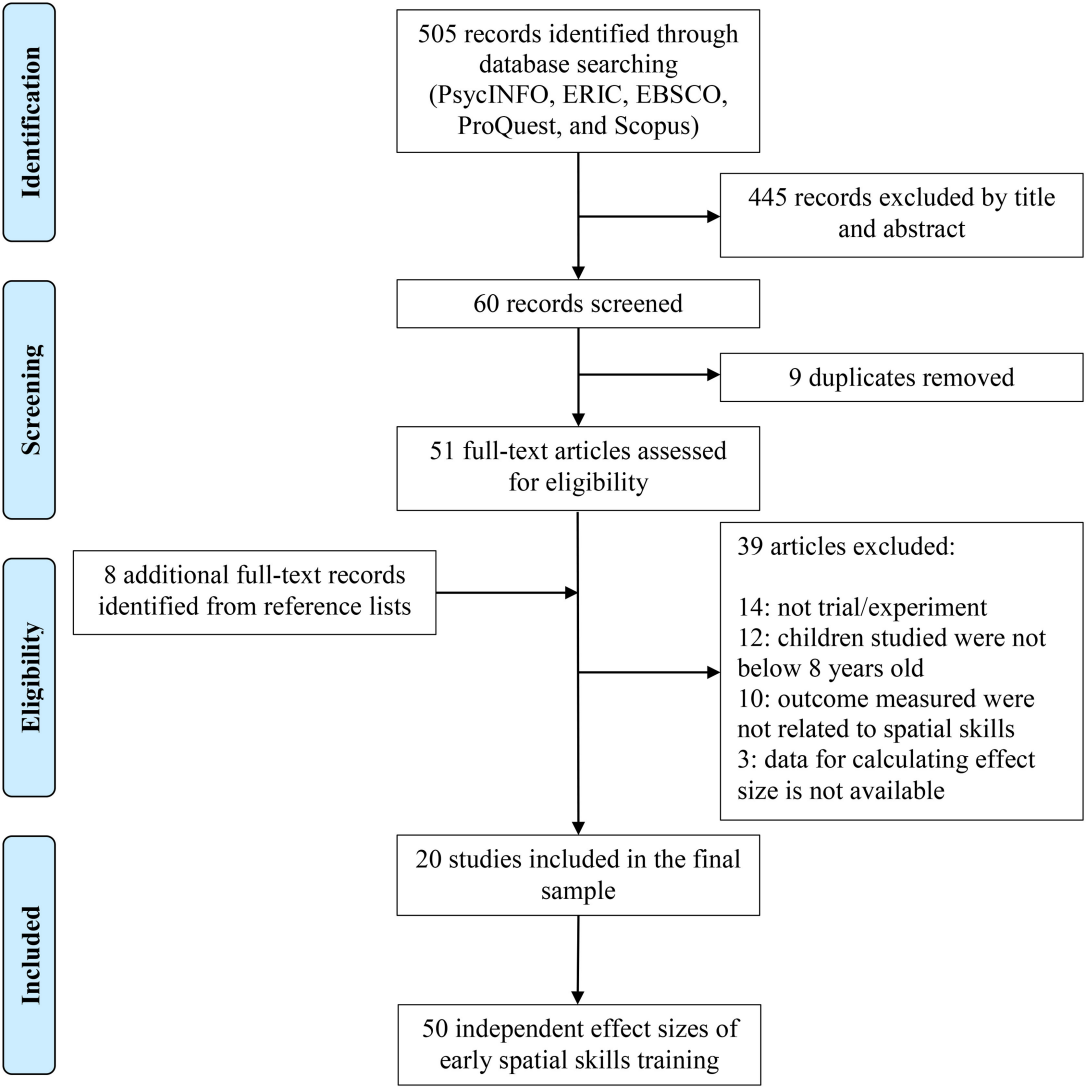
PRISMA diagram of included studies in the meta-analysis.

As shown in Figure 1, which follows the PRISMA statement (Moher et al., 2009), of the 505 records initially identified, 445 were excluded by title and abstract based on the predefined inclusion and exclusion criteria. Of the 60 records remaining and screened, nine were duplicates. We then performed manual searches of the reference list of eligible research reports and repeated this process until no other studies were found, thus adding eight full-text articles. Twenty studies were eventually included, resulting in 50 independent effect sizes.

### Data Extraction

To identify interesting variables for research synthesis, Lipsey (2009) proposed three groups of study descriptors: extrinsic variables, method variables, and substantive variables.

1. Extrinsic variables are represented by fixed characteristics of the study, such as the date of publication, publication type, and funding source. We coded the date of publication in this meta-analysis.
2. Method variables are related to the control of the implementation fidelity and the psychometric properties of the measures. We included the type of study design and the category of spatial skills measures as the two method variables for the moderator analysis.
3. Substantive variables are related to subjects (e.g., sex and age), treatments, and settings. In the current meta-analysis, sex, age, type of training, and research settings represent examples of substantive variables.

To ensure coding reliability, two researchers (the first two authors) independently reviewed a subset (25%) of the articles and used a predefined coding scheme to extract the respective data. The coding scheme addressed the following characteristics of each study: the authors, publication year, sample size, participants' age and sex, types of spatial skills training, categories and measures of children's spatial skills, training settings, study design, and performance of children's spatial skills (effect sizes). After verifying the data coding results, the two researchers showed a high degree of agreement (86%) on all coding items in the subset. The inter-coder reliability (Cohen's kappa) is 0.72, which is considered substantial. Any inconsistencies were resolved through discussion and consensus. The first author finished coding the rest of the articles (75%).

### Data Analyses

We used the Comprehensive Meta-Analysis Version 3 (CMA v3; Borenstein et al., 2013) statistical software package to compute and analyze all the meta-analytic data, as follows:

#### Computing Effect Sizes

We calculated the effect sizes using Hedges's *g*, as the sample sizes in the included studies were mostly small (below 50) (Cohen, 2013; Hedges and Olkin, 2014). This metric is appropriate, as it corrects biases due to sample size (Cohen, 2013). The coefficient of Hedges's *g* represents the difference in means between the two groups relative to the pooled and weighted standard deviation (Cohen, 2013). One effect size was calculated for each outcome category in each study.

Since the data for this meta-analysis were obtained from a series of published studies conducted by different people, it is unlikely that all studies are functionally identical (Borenstein et al., 2007, 2011). In this case, it is suggested that the random effects model is a more reasonable option for the meta-analysis (Borenstein et al., 2007, 2010, 2011). However, when the number of studies is small (*N* < 10), the variance estimate between the studies is usually low, so it is better to calculate the average difference according to the fixed effect model (Borenstein et al., 2010). Therefore, this meta-analysis used a random effects model to calculate the overall effect size and chose either the random effects approach (*N* ≥ 10) or the fixed effect approach (*N* < 10) to calculate and compare the effect sizes across studies involving different categories of outcomes in the moderator analyses.

#### Publication Bias

**Figure 2:**
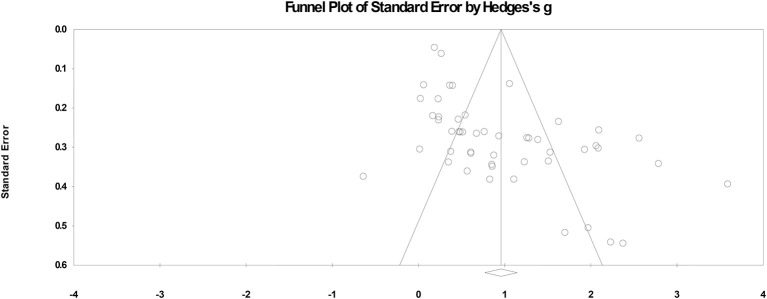
Funnel plot of 50 effect sizes (Hedges' g) generated from included studies to measure for publication bias.

We verified the possibility of publication bias using the trim-and-fill method and a funnel plot of standard error by Hedges's *g* (Duval and Tweedie, 2000). The trim-and-fill analysis only slightly reduced the estimated average effect sizes. The estimated mean values of the trim-and-fill analyses were all significantly different from zero. The results of the additional analysis did not find any variable that could be used as an alternative interpretation of the current results. In addition, a funnel plot was generated against the results to examine the effect size distribution relative to the sample sizes (see Figure 2). Since most of the studies were symmetrically distributed around the average effect size, there was little publication bias observed (Borenstein et al., 2009). Therefore, we report the combined results of the 20 studies and 50 effect sizes in this meta-analysis.

#### Analyzing Variance in Effect Sizes

We studied the variability of the effect sizes across studies through the heterogeneity test (Hedges and Olkin, 2014; Schmidt and Hunter, 2014; Cooper, 2016). We thus identified moderators that may not have been studied in a single experiment and that may affect the magnitude of the training effects (Cooper, 2016).

A heterogeneity test compares the variance shown by a set of effects with the assumed variance due to sampling error (Higgins et al., 2003; Cooper, 2016). If the heterogeneity test results indicate that the difference in a set of effects can be attributed only to the sampling error, then the data can be assumed to represent the population of participants (Hunter et al., 1982). We used the inter-group statistic, *Q*, to assess whether the group average effect is homogeneous (Yang et al., 2019). A statistically significant *Q* indicates that the grouping factor contributes to the variance in effect size; in other words, the grouping factor has a significant effect on the measurement of outcomes (Higgins et al., 2003).

## Results

### Effects of Early Spatial Interventions

We meta-analyzed 20 intervention studies on spatial skills for children aged 0–8 years. There were 900 children in the training group and 635 children in the control group. Table 2 presents the effect sizes and key characteristics of the included studies.

**Table 2:** Effect sizes and key characteristics of studies included in the meta-analysis.

**Study (year)**	**Training description**	**Training category^a^**	**Training setting^b^**	***N* of children (T/C)**	**Effect size (Hedges's *g*)**	**Study design^c^**	**Outcome measure**	**Outcome category^d^**	**Age^e^**	**Sex^f^**
Frick et al. (2009): overall		1	3 (school but not the original classroom)	32	0.311	1	Water tilting task	5	2	1, 2
Frick et al. (2009): treatment 1	Visibly executed movement in the water tilting task (manual tilting task)									
Frick et al. (2009): treatment 2	Seeing but not executing movement in the water tilting task (visible but regulated by means of remote control tilting task)									
Frick et al. (2009): treatment 3	Executing but not seeing movement in the water tilting task (blind tilting task)									
Frick et al. (2009): control	Not perceiving any movement in the water tilting task (static judgment task)									
Tzuriel and Egozi (2010): overall	Visuospatial representation and transformation program based on Quick Draws activities	2	2	60/56	0.582	3	PMA—Spatial Relations (SR) subtest; WT	2	2	1, 2
Ping et al. (2011): overall		1	1		2.066	3	CMTT; MROT	2	2	1, 2
Ping et al. (2011): treatment 1	Using gesture to rotate objects on a computer screen			22						
Ping et al. (2011): treatment 2	Turning a joystick to rotate objects on a computer screen			20						
Ping et al. (2011): control	No training			21						
Goldin-Meadow et al. (2012): overall	Performing a Move gesture as compared to observing a Move gesture	3	1	78/80	0.211	3	Mental transformation task (piece cards and choice card)	2	2	1, 2
Keren et al. (2012): overall	Playing with Kindergarten Assistive Robotics (KAR) through a musical game	1	2	9	1.539	1	Acquisition of spatial–motor knowledge measured using a metaphor of movement velocity	5	2	1, 2
Nachtigäller et al. (2013): overall	Comprehending the preposition UNDER with six object sets, with the word UNDER embedded in a narrative context	3	1	20/20	0.386	3	Performance of the spatial relations UNDER and ON	3	1	1, 2
Chen et al. (2013): overall		2	3 (medical center)		0.655	3	TVPS-3	3	2	1, 2
Chen et al. (2013): treatment 1	Multimedia visual perceptual group training program			15						
Chen et al. (2013): treatment 2	Multimedia visual perceptual individual training program			15						
Chen et al. (2013): treatment 3	Paper visual perceptual group training			19						
Chen et al. (2013): control	No visual perceptual training			15						
Möhring and Frick (2013): overall	Manual exploration of the object	1	1	20/20	0.909	2	Mental rotation test (looking time)	2	1	1, 2
Henry et al. (2014): overall		2	3 (school but not the original classroom)		0.862	3	Odd One Out Span	1	2	3
Henry et al. (2014): treatment	10 min working memory intervention tasks, three times a week, for a total of 6 weeks			18						
Henry et al. (2014): control	Equal one-to-one attention but simpler versions of the tasks, with no requirement for memory storage			17						
Chabani and Hommel (2014): overall	Tangram problem solving with visual prompts	3	1	99/94	0.282	3	Tangram puzzles	5	2	1, 2
Frick and Wang (2014): overall	Acting upon the turntable themselves (self-turning condition)	1	1	14/14	0.091	2	Sensitivity to spatial object relations (mean looking times)	2	1	1, 2
Hawes et al. (2015): overall	Computerized mental rotation games (playing three games that were all housed within an application in iPad)	1	2	32/29	1.297	3	CMTT; Visual–Spatial Puzzle Task; tests of 2D and 3D mental rotation	2	2	1, 2
Metin and Aral (2016): overall	Project-based education for supporting visual perception	2	2	22/22	1.519	3	MVPT-3	3	2	1, 2
Xu and LeFevre (2016): overall	Non-numerical spatial training (i.e., decomposition of shapes)	3	3	42/42	0.553	3	2D mental transformation task	2	2	1, 2
Hawes et al. (2017): overall	A 32-week teacher-led spatial reasoning intervention (i.e., geometry lessons and quick challenge spatial activities)	2	2	39/28	2.702	3	Spatial language test; visual–spatial geometry test; CMTT	2	2	1, 2
Borriello and Liben (2018): overall	Conversational instructions for guiding parents to engage their children in spatial play	1	1	19/22	0.496	2	Spatial language coded	5	2	1, 2
Levine et al. (2018): overall		3	3 (school but not the original classroom)		0.359	3	Mental transformation task (piece cards and choice card)	2	2	1, 2
Levine et al. (2018): treatment 1	Making a motor movement that is relevant to the mental transformation through action (concrete training)			41						
Levine et al. (2018): treatment 2	Making a motor movement that is relevant to the mental transformation through gestural movements (abstract training)			38						
Levine et al. (2018): control	Point-gesture training			35						
Yeterge et al. (2019): overall	Creative drama as an approach to sensory integration education	2	2	17/17	0.867	3	FVPT	1	2	3
Cornu et al. (2019): overall	A tablet-based visuospatial intervention, with many different tasks targeting different aspects of visuospatial skills	2	2	68/57	0.136	3	Spatial orientation measure adapted from FVPT; CMTT	1, 2	2	1, 2
Bower et al. (2020): overall	Constructing puzzles to match a model composed of various geometric shapes	3	3 (school but not the original classroom)		2.053	3	2D TOSA; 3D TOSA	2	2	1, 2
Bower et al. (2020): treatment 1	Giving modeling and feedback			46						
Bower et al. (2020): treatment 2	Giving gesture feedback			48						
Bower et al. (2020): treatment 3	Giving spatial language feedback			47						
Bower et al. (2020): control	No feedback			46						

*T, training group; C, control group; CMTT, Children's Mental Transformation Task; MROT, Mental Rotation Task; TVPS-3, Test of Visual Perception Skills, 3^rd^ Edition; PMA, Primary Mental Abilities; WT, Windows Test; CMTT, Children's Mental Transformation Task; MVPT, Motor-Free Visual Perception Test, 3^rd^ Edition; FVPT, Frostig Visual Perception Test; TOSA, Test of Spatial Assembly*.

a *1 = video game/play/hands-on operation; 2 = classroom-based course; 3 = spatial task training*.

b *1 = lab; 2 = classroom; 3 = others*.

c *1 = within subjects; 2 = between subjects; 3 = mixed*.

d *1 = intrinsic, static; 2 = intrinsic, dynamic; 3 = extrinsic, static; 4 = extrinsic, dynamic; 5 = measure that spans cells*.

e *1 = 0–3 years; 2 = 4–8 years*.

f *1 = female; 2 = male; 3 = not specified*.

As shown in Table 2, previous studies used different types of training to promote young children's spatial skills, including video games, play, hands-on operation, classroom-based courses, and specific spatial tasks. Among the 20 intervention studies, 35% (*N* = 7) used video games, play, or hands-on operation for training; 35% (*N* = 7) used classroom-based courses; and 30% (*N* = 6) used specific spatial tasks. In terms of the setting where the training took place, 35% (*N* = 7) of training programs were conducted in a lab, with 35% (*N* = 7) conducted in the children's original classroom and 30% (*N* = 6) in other places such as another room in their preschool settings. All studies sampled children aged 0–8, with 15% (*N* = 3) of them being infants and toddlers (0–3 years) and 85% (*N* = 17) in early childhood (3–8 years). Study design and measurement of children's spatial skills also varied across studies, with details presented in Table 2.

Although publication biases always exist in any meta-analysis (Lipsey and Wilson, 1993) (see the funnel plot in Figure 2), the random effects analysis results revealed that the average effect size (Hedges's *g*) for training relative to control was 0.96 (*SE* = 0.10).

### Moderator Analyses

We further analyzed the moderating effects of several study descriptors, including the type of study design, sex, age, outcome category (i.e., type of spatial skills), research setting (e.g., lab vs. classroom), and type of training. We used the *Q* statistic to assess the significance of the heterogeneity test in the effect size. Table 3 presents the results of the moderator analysis of the effects of these six study descriptors on the spatial skills of the participating children.

**Table 3:** Heterogeneity tests of effect sizes (Hedges's g) for potential moderators.

**Potential moderators**	***Q***	***N***	***g***	***SE***
Study design^a^	61.830^*^			
Within subjects		5	0.328^*^	0.037
Between subjects		7	0.529^*^	0.126
Mixed		38	0.759^*^	0.041
Sex^a^	9.405^*^			
Girls		5	0.909^*^	0.143
Boys		5	0.686^*^	0.141
Not specified		40	0.499^*^	0.028
Age^a^	0.000			
0–3 years		5	0.518^*^	0.153
4–8 years		45	0.520^*^	0.027
Spatial skills outcomes^a^	111.263^*^			
Intrinsic–static		3	0.456^*^	0.145
Intrinsic–dynamic		31	0.952^*^	0.050
Extrinsic–static		5	0.770^*^	0.154
Measure that spans cells		11	0.326^*^	0.033
Research setting^b^	4.229			
Lab		17	0.690^*^	0.169
Classroom		19	1.158^*^	0.155
Others		14	0.989^*^	0.177
Type of training^b^	1.673			
Video game/play/hands-on operation		21	1.069^*^	0.156
Classroom-based course		17	0.993^*^	0.171
Spatial task training		12	0.752^*^	0.194

N, number of effect sizes; SE, standard error;

* *p < 0.01*.

a *Fixed effect approach is used for the moderator analysis of this variable*.

b *Random effects approach is used for the moderator analysis of this variable*.

As shown in Table 3, the type of study design [within subjects (*g* = 0.328) < between subjects (*g* = 0.529) < mixed (*g* = 0.759)], sex [girls (*g* = 0.909) > boys (*g* = 0.686) > mixed (*g* = 0.499)], and outcome category [generic (*g* = 0.326) < intrinsic, static (*g* = 0.456) < extrinsic, static (*g* = 0.770) < intrinsic, dynamic (*g* = 0.952)] were found to moderate the training effects. However, there was no significant difference in age, type of training, and research setting as related to children's spatial skills outcomes.

## Discussion

Although existing meta-analyses have demonstrated that spatial skills are malleable and can be improved by training (Baenninger and Newcombe, 1989; Uttal et al., 2013), none of them exclusively focuses on the effect of training on young children's spatial skills. To the best of our knowledge, this meta-analysis is the first attempt of its kind to systematically review and investigate the effects of spatial skills training in children aged 0–8 years.

### Early Intervention Matters in the Development of Spatial Skills

This meta-analysis revealed that diverse training strategies or programs including hands-on exploration, visual prompts, and gestural spatial training could significantly foster young children's spatial skills. This finding demonstrated that young children's spatial skills could be significantly improved if they are given specific training, with an average effect size (Hedges's *g*) of 0.96 for training relative to control. The effect size obtained in the current meta-analysis is greater than the average effect (*g* = 0.47) indicated in Uttal et al.'s (2013) results. Therefore, our finding seems to support the argument that spatial skills, a kind of cognitive trait, are more malleable in the early years of life than the later stages such as adolescence and adulthood. However, this argument warrants further investigation, as only published papers are included in this meta-analysis, and publication bias may exist (Thornton and Lee, 2000).

The positive effect of early spatial skills training revealed in this study aligned with the theoretical links between action and cognition for understanding the underlying mechanism of effective early spatial training strategies or programs. According to Newcombe and Frick (2010), mental rotation and spatial perspective taking are the most crucial precursory forms of spatial skills in the early years, which are commonly related to motor development. Motor activities can thus facilitate children's performance in mental rotation and spatial perspective-taking tasks by engaging them in active movement (Newcombe and Frick, 2010). As found in the present meta-analysis, most of the effective spatial training used video games, play, hands-on exploration, spatial tasks, or classroom-based courses as the intervention or stimuli. What they have in common is that hands-on exploration, visual prompts, and gestures are used to support the process of actively practicing spatial skills in various activities (e.g., Frick et al., 2009; Borriello and Liben, 2018; Bower et al., 2020). It is possible that the engagement in manipulating visuospatial information would require the involvement of different neural processes. This could further shape the neural functioning related to spatial skills. However, the neural mechanism has not yet been thoroughly unveiled in spatial training studies and requires more future research to make sense of the positive effects of early intervention on children's spatial skills.

### Differences in the Response to Training: Study Design, Sex, and the Category of Spatial Skills

Our results revealed that the type of study design, sex, and outcome category moderated the effects of early spatial skills training. However, the moderator analyses revealed that age, research setting, and type of training did not have a significant, moderating effect on the training outcomes. The combined effect sizes indicated that different groups of age, training settings, and training approaches did not generate significantly different effect in promoting young children's spatial functioning. These findings suggest that various approaches such as hands-on exploration, visual prompts, and gestural spatial training could all lead to improvements in spatial skills across different age groups in the early years. This aligns with theoretical arguments given by Ehrlich et al. (2006) that environmental input plays a crucial role in the development of spatial skills, even though biology also contributes to spatial skills.

As revealed in this meta-analysis, research setting did not play a moderating role; however, as argued by Klahr and Li (2005), there is an urgent need for studies on integrating cognitive research in laboratories with teaching in classrooms. A recent experimental study conducted by Hawes et al. (2017) provided empirical evidence that a classroom-based spatially enriched geometry course with a relatively long duration of 32 weeks could lead to young children's considerable progress in spatial skills. This research agenda requires more attention and endeavors, as our evidence indicated that classroom-based spatial skills training might be more effective (*g* = 1.16 > 0.69 in the laboratory setting). Below we further discuss the confirmed moderating factors.

#### Study Design

This meta-analysis revealed that the study design quality moderated the training effects. Although we only included studies using a (quasi-)experimental design, there are three different levels of quality regarding the rigor of design. The results showed that those experiments with both a within- and between-subjects design (*N* = 38) had the largest effect sizes regarding the training effect (average *g* = 0.759). However, it is unclear why within-subject comparison does not lead to a higher extent of positive training effect on average. There are two possible explanations. First, this may be caused by the effect of publication bias, as academic journals tend to be in favor of between-subject experimental research with more positive results (Song et al., 2010). Second, the existence of a control group seems to increase the effect sizes of training; therefore, we suggest that there could be negative effects brought by the lack of targeted spatial skills training for specific assessments. As this may be contradictory to the potential learning of test-taking strategies by children in the control group (Müller et al., 2012), more research is needed to directly investigate these claims regarding the effect in the control group such as practice effects in spatial skills assessments among young children.

#### Sex

Existing meta-analyses demonstrated that men outperform women on measures of mental rotation and spatial perception (Linn and Petersen, 1985; Voyer et al., 1995; Maeda and Yoon, 2013). The male performance advantage in spatial skills seems to start as early as infancy and early childhood (Levine et al., 1999; Moore and Johnson, 2008; Quinn and Liben, 2008). Our meta-analysis revealed that early spatial skills training would lead to greater effect for girls (*g* = 0.909) than boys (*g* = 0.686). Our finding supports the suggestion given by Newcombe and Frick (2010) that the integration of spatial learning opportunities into early childhood education could not only promote spatial skills in general but also reduce early sex differences that may impede female citizens' full participation in the current digital world. Such an encouraging consequence of introducing spatial skills training in early childhood settings further demonstrates that experiences with spatially enriched stimuli and activities would benefit children in their spatial cognition and reduce the sex differences in this cognitive trait (Baenninger and Newcombe, 1989; Moore and Johnson, 2008).

#### Category of Spatial Skills Assessment

This meta-analysis revealed that the category of spatial skills measures moderated the training effects. Results indicated that different categories of spatial tasks respond differently to training, with the mean weighted effect sizes for intrinsic–static, extrinsic–static, and intrinsic–dynamic kinds of assessment at 0.456, 0.770, and 0.952, respectively. The moderating role of the kinds of spatial skills assessment is consistent with the result revealed in Uttal et al.'s (2013) meta-analysis. However, in the early years, children tended to perform better in mental rotation as featured in the intrinsic–dynamic category of assessment instead of the extrinsic–static category. Although our finding seems to align with an extensive body of literature that records infants' and young children's performance in mental rotation tasks (e.g., Moore and Johnson, 2008; Frick and Wang, 2014; Lehmann et al., 2014), more direct research is needed to ascertain what the exact differences of effects are when measuring children's spatial skills using different assessments.

### Limitations of This Meta-Analysis

One of the limitations of our meta-analysis is that as the number of studies involved is relatively small, the effect sizes across studies are considerably heterogeneous. The variance in effect size may explain why heterogeneity between groups is not significant for the results of moderator analyses of certain research descriptors (e.g., type of training and research setting). Although the publication biases were shown to be acceptable using the trim-and-fill method, the generalization of our findings to other contexts and populations should be conducted with caution due to the small number of eligible studies included. Moreover, only published English papers were included in this meta-analysis due to the inaccessibility of other types of articles. This may have led to biases in our meta-analysis because studies reporting a significant impact are more likely to be published than studies not reporting statistical significance (Rosenthal, 1979).

Also, our moderator analyses did not cover the factors of SES, initial level of performance on spatial tasks, and intervention duration. The included studies reported that their participants were from families of diverse socioeconomic backgrounds; therefore, we were not able to analyze the moderating effect of SES in the current meta-analysis. Although this meta-analysis attempted to control study design, it was still unable to adequately capture or control certain variables, such as trainers' qualifications and the duration of training, because these variables were not clearly reported in the included studies.

Last but not least, this meta-analysis did not include non-experimental research as well as those studies on transfer effects of spatial skills training to untrained tasks. The current meta-analysis only included studies examining the relationship between training programs and the development of spatial skills. However, meta-regression can also be used to examine the relationship between spatial training and children's spatial skills and other related outcomes (e.g., math skills, scientific task performance, and executive function), so that correlational studies can be meta-analyzed. Correlational studies may be valuable for exploring the complex behavioral and neural mechanisms behind the training effect. Subsequent qualitative systematic reviews or meta-regression analyses of the processes and mechanisms through which early spatial skills can be enhanced would be of great importance.

### Implications for Research, Policy, and Practice

Our research contributes to the literature in the field of spatial thinking by showing whether and how early intervention approaches and programs can promote young children's spatial functioning through meta-analytic evidence. Our meta-analysis thus expands this line of research on the malleability of spatial skills in the early years and provides the following implications for future research, policy-making, and practice in early childhood education.

First, early spatial intervention matters. Our evidence indicated that the malleability of spatial skills is stronger in younger children, as compared to the average effect size (*g* = 0.47) found in the general population (Uttal et al., 2013).

Second, a spatially enriched curriculum should play a more vital role in early childhood education via the integration of effective practices such as spatial play (block building) and purposeful use of visual and verbal cues. This is also supported by our evidence that classroom-based spatial skills training is more effective (*g* = 1.16) than laboratory-based training (*g* = 0.69). To implement effective spatially oriented curricula in early childhood settings, more specific research is needed to design, implement, and evaluate classroom-based spatial training programs for young children.

Third, as linked to the previous implication, both early childhood policymakers and practitioners should consider scaling up effective classroom-based spatial training. Publicity and promotion require not only more research endeavors but also initiatives in policy and practice so as to bridge the gap between the laboratory environment and authentic learning settings and foster early spatial skills among children from diverse backgrounds, especially those placed in socially disadvantaged environments such as poverty and adverse parenting practices.

Fourth, to support children with difficulties in spatial functioning, spatially relevant game tasks can be used. For instance, visuospatial representation and transformation activities based on Quick Draws (Tzuriel and Egozi, 2010), playing with robotics (Keren et al., 2012), rotating objects on mobile devices or computers (Ping et al., 2011; Hawes et al., 2015; Cornu et al., 2019), and tangram-related activities (Chabani and Hommel, 2014) are shown to significantly foster young children's spatial skills. Moreover, adult educators such as teachers and parents can provide children with more opportunities of manual exploration of the object, such as building blocks (Möhring and Frick, 2013), and intentionally give various types of feedback (e.g., modeling, gesture feedback, and spatial language feedback) during spatially relevant activities (Bower et al., 2020). Some early interventions such as a multimedia visual perceptual individual training program (Chen et al., 2013) and spatial reasoning intervention including geometry lessons and quick challenge spatial activities (Hawes et al., 2017) can also be provided. However, more studies are needed to explore how to tailor spatial training programs to the specific abilities and disabilities of individual children.

Fifth, as early spatial skills training is demonstrated to more effectively enhance girls' spatial functioning and minimize the male advantage in this aspect, girls should be given the priority to engage in spatially enriched experiences.

Last but not least, more future research is warranted to explore the behavioral and neural mechanisms underlying the effects of spatial training in the early years. Two aspects should be focused on: study design and assessment. On the one hand, future research should draw upon a more rigorous design using randomized controlled trials and even a longitudinal design to investigate the training effects in the long run. One the other hand, there is an urgent need to conduct specific research on measuring children's spatial skills using different assessments. Moreover, how the improvement of early spatial skills may be linked to fostering other core skills such as numeracy, math reasoning, early writing skills, and executive functions can be explored in the future.

## Conclusion

This meta-analysis supports the notion that effective spatial learning components could be infused into early childhood settings, so as to spatialize the curriculum and encourage children learn to think spatially (Newcombe and Frick, 2010; Bruce et al., 2015). To implement effective spatially oriented curricula in early childhood settings (Newcombe and Frick, 2010; Uttal and Cohen, 2012), early childhood researchers, policymakers, and practitioners should work together to intentionally support children's hands-on, proactive manipulation and processing of spatial information. The US National Research Council (2006) has released a national report to call for a curriculum and support system for spatial thinking in the K-12 educational context. Taking off from this research and policy achievement, high-quality, evidence-based, contextually appropriate spatial curricula should also be developed and provided for children to promote their spatial intelligence and help them become better prepared for the high-tech world.

## Author Contributions

WY designed the research and drafted the manuscript. WY, HL, NC, and PX collected and extracted data for analysis. XL provided important ideas and substantial feedback for the study and edited the manuscript. All of the authors read and approved the final manuscript.

## Conflict of Interest

The authors declare that the research was conducted in the absence of any commercial or financial relationships that could be construed as a potential conflict of interest.
